# Autophagy and Tau Protein

**DOI:** 10.3390/ijms22147475

**Published:** 2021-07-12

**Authors:** Tadanori Hamano, Soichi Enomoto, Norimichi Shirafuji, Masamichi Ikawa, Osamu Yamamura, Shu-Hui Yen, Yasunari Nakamoto

**Affiliations:** 1 Second Department of Internal Medicine, Faculty of Medical Sciences, University of Fukui, Eiheiji-cho, Fukui 910-1193, Japan; weltraum@u-fukui.ac.jp (S.E.); sira@u-fukui.ac.jp (N.S.); iqw@u-fukui.ac.jp (M.I.); kapi@u-fukui.ac.jp (O.Y.); nakamoto-med2@med.u-fukui.ac.jp (Y.N.); 2 Department of Aging and Dementia (DAD), University of Fukui, Eiheiji-cho, Fukui 910-1193, Japan; 3 Life Science Innovation Center, University of Fukui, Eiheiji-cho, Fukui 910-1193, Japan; 4 Mayo Clinic Jacksonville, Jacksonville, FL 3224, USA; yen.shu-hui@mayo.edu

**Keywords:** autophagy, Alzheimer’s disease, tau protein, amyloid β protein, autophagic vacuoles, mTORC1

## Abstract

Neurofibrillary tangles, which consist of highly phosphorylated tau protein, and senile plaques (SPs) are pathological hallmarks of Alzheimer’s disease (AD). In swollen axons, many autophagic vacuoles are observed around SP in the AD brain. This suggests that autophagy function is disturbed in AD. We used a neuronal cellular model of tauopathy (M1C cells), which harbors wild type tau (4R0N), to assess the effects of the lysosomotrophic agent NH4Cl, and autophagy inhibitors chloroquine and 3 methyladenine (3MA). It was found that chloroquine, NH4Cl and 3MA markedly increased tau accumulation. Thus, autophagy lysosomal system disturbances disturbed the degradation mechanisms of tau protein. Other studies also revealed that tau protein, including aggregated tau, is degraded via the autophagy lysosome system. Phosphorylated and C terminal truncated tau were also reported to disturb autophagy function. As a therapeutic strategy, autophagy upregulation was suggested. Thus far, as autophagy modulators, rapamycin, mTOCR1 inhibitor and its analogues, lithium, metformin, clonidine, curcumin, nicotinamide, bexaroten, and torehalose have been proposed. As a therapeutic strategy, autophagic modulation may be the next target of AD therapeutics.

## 1. Introduction

**Figure 1:**
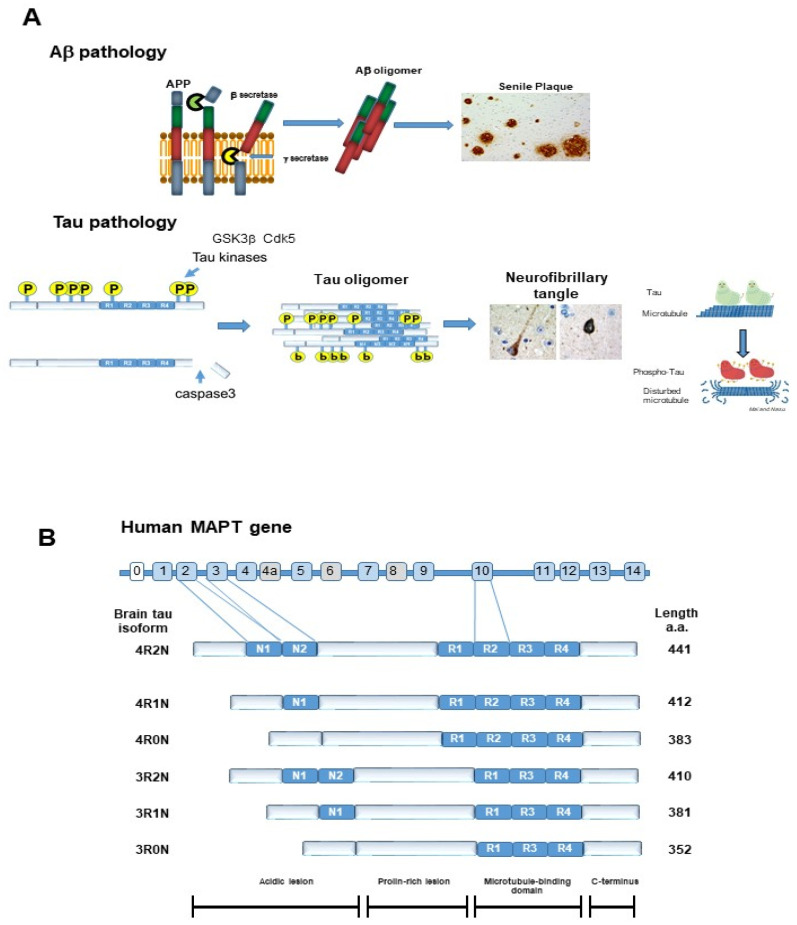
Pathological hallmarks of Alzheimer’s disease. (**A**) Amyloid β protein (Aβ) was generated by cleaving amyloid precursor protein (APP) with β secretase and γ secretase. Aβ forms oligomers and finally forms senile plaques. Once tau protein is hyper-phosphorylated by tau kinases, including GSK3β or cdk5, it detaches from the microtubule and forms aggregates, called tau oligomers. C terminal-truncated tau by caspase3 and N-terminal truncation accelerates tau aggregation. Tau oligomers are neurotoxic and further aggregation leads to neurofibrillary tangles (NFT). (**B**) Tau isoforms and phosphorylation epitopes. There are 6 tau isoforms according to the number of N terminal insertions and repeats of the microtubule binding domain: 4 repeat 2 N (4R2N), 4 repeat 1N (4R1N), 4 repeat 0N (4R0N), 3 repeat 2N (3R2N), 3 repeat 1N (3R1N), and 3 repeat 0N (3R0N).

Neurofibrillary tangles (NFT), which consist of extensively phosphorylated tau, and senile plaques (SP), which consist of amyloid β protein (Aβ), are pathological hallmarks of Alzheimer’s disease (AD). Tau protein stabilizes the microtubule [1] and is important for axonal transport [2]. However, once tau is highly phosphorylated, it undergoes a conformational change and can no longer bind microtubules. The free tau forms aggregates, known as tau oligomers (Figure 1). Further aggregation of tau oligomers forms NFT. Tau oligomers are more toxic to neurons than NFT [3]. AD, progressive supranuclear palsy, corticobasal degeneration, argyrophilic grain disease, Pick’s disease, frontotemporal dementia with parkinsonism linked to chromosome 17 (FTDP-17), Niemann–Pick disease type C, and chronic traumatic encephalopathy are collectively known as tauopathies as they have common characteristics such as the pathological tau accumulation [4,5]. 

There are several factors that cause tau aggregation. First is the activation of tau kinases, including glycogen synthase kinase 3β (GSK3β), cyclin-dependent kinase5 (cdk5), P38 mitogen-activated protein kinase (P38MAPK), and stress-activated protein kinase (SAPK)/Jun amino terminal kinase (JNK), or inactivation of tau phosphatase, protein phosphatase 2A (PP2A). Second, tau cleavage at C terminus can be the factor that accelerates tau aggregation because the C terminus of tau inhibits tau aggregation (Figure 1A) [6]. Another important factor that leads to tau aggregation is the disturbance of tau clearance. As the mechanisms of tau clearance, two pathways have been proposed, the ubiquitin proteasome system and the autophagy lysosome system. 

In this review, we describe the process by which the autophagy lysosome system degrades tau protein, including aggregated tau, and the possibility of autophagy regulation as a therapeutic strategy of tauopathies, including AD.

## 2. Tau Protein

### 2.1. Physiological Function of Tau

The tau gene is located on chromosome 17. There are 6 isoforms by alternative splicing of exons 2, 3, and 10. Isoforms have different numbers of N-terminal insertions (0, 1, or 2) and microtubule-binding repeat domains (3R, or 4R), which produce 6 isoforms, 4 repeat 1N (4R1N), 4 repeat 2N (4R2N), 4 repeat 0N (4R0N), 3 repeat 1N (3R1N), 3 repeat 2N (3R2N), and 3 repeat 0N (3R0N) (Figure 1B). The major role of tau is widely considered to be microtubule stabilization [1] and it has an essential role in axonal transport [2]. However, the interaction of tau with microtubules is intermittent and short, and is metaphorically referred to as a rapid “kiss and hop process” [7]. Many studies demonstrate that tau can regulate multiple signaling pathways. For example, tau facilitates insulin-induced tyrosine phosphorylation of the insulin receptor substrate 1 (IRS-1) and inactivates phosphatase and tensin homolog (PTEN). In conclusion, the essential physiological roles of tau in the brain remain unclear [8]. 

Of note, experimental decrease or removal of tau in vivo does not change various neural properties or processes that are considered dependent on microtubules, such as neuronal integrity, axonal transport, synaptogenesis, and complex brain functions. Recently, the decrease of tau was reported to be essential for the treatment of tauopathies [8,9].

### 2.2. Post-Translational Modification of Tau

#### 2.2.1. Phosphorylation of TAU PROTEIN

**Figure 2:**
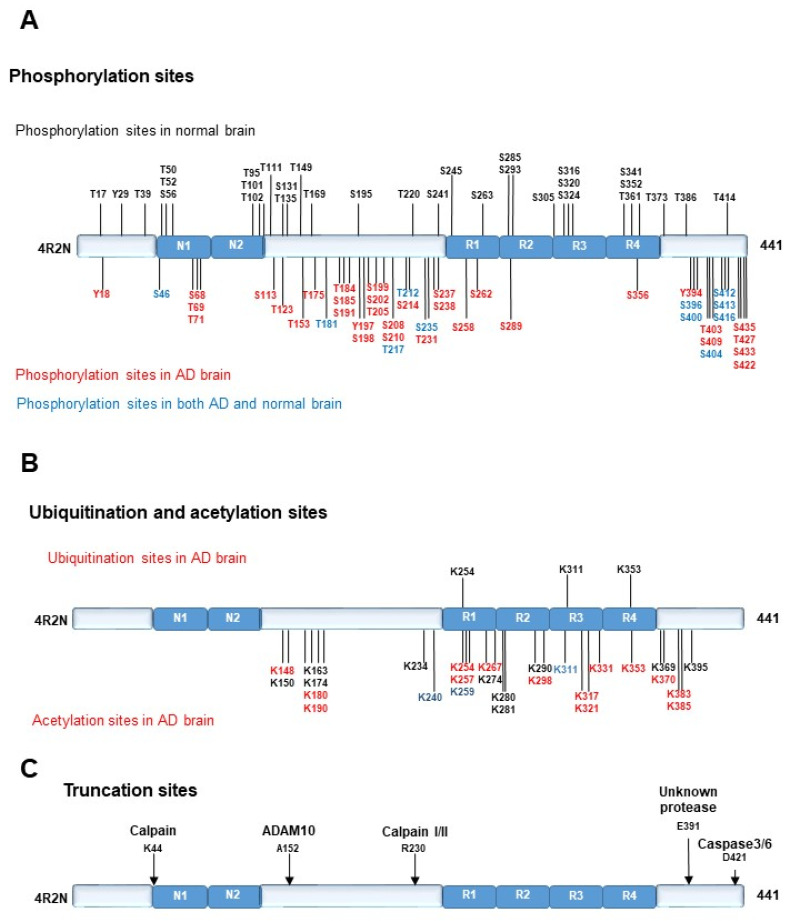
Post-translational modification of tau protein. (**A**) Putative phosphorylation sites of tau protein. Red color shows amino acids phosphorylated in AD brain, blue color in AD and normal brain, and black color in normal brain [10]. (**B**) ubiquitination sites (upper) observed in AD brain, and acetylation by p300 and CREB-binding protein (CBP) (black), only by p300 (red), or only by CBPblue) are also shown [15]. (**C**) Truncation sites of tau protein. The sequential truncation of tau can produce tau fragments. The cleavage of tau is more likely to happen when tau is detached from microtubules, either forming aggregates of each other, or becoming associated with proteins other than tubulin [10,17].

Tau is phosphorylated at several sites by multiple tau kinases [8,10]. The phosphorylation sites in the normal brain and AD brain are shown in Figure 2A. The levels of phosphorylation of tau at Ser199, Ser202/Thr205, and Ser422 were comparable in healthy controls and Braak stage I-IV AD patients, but increased in Braak stage V/VI in the isocortex and transentorhinal cortex. Only tau phosphorylation at Tyr18 and Thr231 markedly increased in the transentorhinal region at Braak Stage III/IV, and further increased with increasing Braak Stage. In addition, the highest increases in phosphorylation compared with controls were noted at Thy18, Thy231, and Ser199. Conversely, Ser396tau and Ser262 tau were only weakly phosphorylated and progressed only slightly in all brain regions analyzed [11].

#### 2.2.2. Ubiquitination of Tau Protein

Ubiquitination is an essential post-translational modification and has an important function in eukaryotic cells such as in protein homeostasis, signal transduction, and subcellular localization. Ubiquitin is attached to the target protein Lyn residue by covalent bonding and functions as a degradation signal by the 26S proteasome. As NFT are also immunostained by antibodies against ubiquitin, paired helical filament (PHF)-tau undergoes ubiquitination. Analysis of AD brain-derived tau revealed that most of the ubiquitinated tau is mono-ubiquitinated and some is polyubiquitinated. In addition, the tau found here was N-terminally truncated. It is thought that excessively phosphorylated tau aggregates as PHF-tau, the N-terminal is truncated, and the tau then undergoes ubiquitination. The C terminus of the Hsc70-interacting protein (CHIP), which directly interacts with Hsp70/90, induces tau ubiquitination. CHIP further increases tau aggregation [12]. The ubiquitination site of tau is shown in Figure 2B. 

#### 2.2.3. Acetylation of Tau Protein

Acetylation is another post-translational modification. Lysine residues are characterized by their involvement in electrostatic and hydrophobic interactions, and have an important role in tau polymerization and toxicity. Therefore, there has been interest in whether acetylation at lysine residues of tau regulates tau polymerization. Cohen et al. found that CREB (cAMP-response element-binding protein)-binding protein (CBP), an acetyltransferase, acetylates a fragment of tau containing a microtubule-binding domain and increases aggregation of that fragment [13]. The acetylation sites are shown in Figure 2B [14]. 

It was recently suggested that acetylated tau inhibits chaperon-mediated autophagy (CMA)-mediated degradation, as described below. Therefore, acetylated tau is thought to be preferentially degraded by macroautophagy and microautophagy of endosomes. However, other reports stated that acetylation of full-length tau by acetyltransferase p300 reduces tau aggregation and that it is reversed by the addition of the deacetylase histone deacetylase (HDAC6) [15]. 

#### 2.2.4. Truncation of Tau Protein 

Tau proteins purified from the core of PHF are truncated at their N- or C-terminal portions. Tau truncation was demonstrated to promote tau aggregation [16,17]. As tau is an unfolded or unfoldable protein by nature, it is easily digested by proteases. Many studies confirmed that tau can be digested by calpains, caspases [6,17,18,19,20], and A disintegrin, and metalloproteinase 10 (ADAM10). Tau is also a substrate for aspartate endopeptidase. These studies suggested that tau cleavage is important for tau aggregation and neurodegeneration [16,17]. Several specific tau cleavages have been identified in the AD brain, such as truncation at Asp421(D421) and Glu391 (E391), which promote tau aggregation [16,17] (Figure 2C). In addition, tau fragments can propagate between neurons via synapses and expand neurofibrillary degeneration to postsynaptic neurons, as described below [10].

#### 2.2.5. Toxicity of Tau

Aberrant phosphorylation, aggregation, and proteolysis of tau protein during the preliminary stages of neurofibrillary degeneration (Figure 2) are neuropathologically important in the development of AD. However, which type of tau is most toxic (misfolded fibrous aggregates, soluble hyperphosphorylated, or mislocalized) and whether the toxicity is due to a gain or loss of function remain unclear. As there is limited direct evidence that tau fibers themselves are toxic, tau in its soluble oligomeric form being more toxic to neurons and synapses is becoming more plausible [10]. In the normal brain, tau is mainly observed in axons [2]. However, in the AD brain, NFTs, composed of highly phosphorylated tau, are observed not only in axons, but also in the cytosol and dendrites. 

## 3. Autophagy

**Figure 3:**
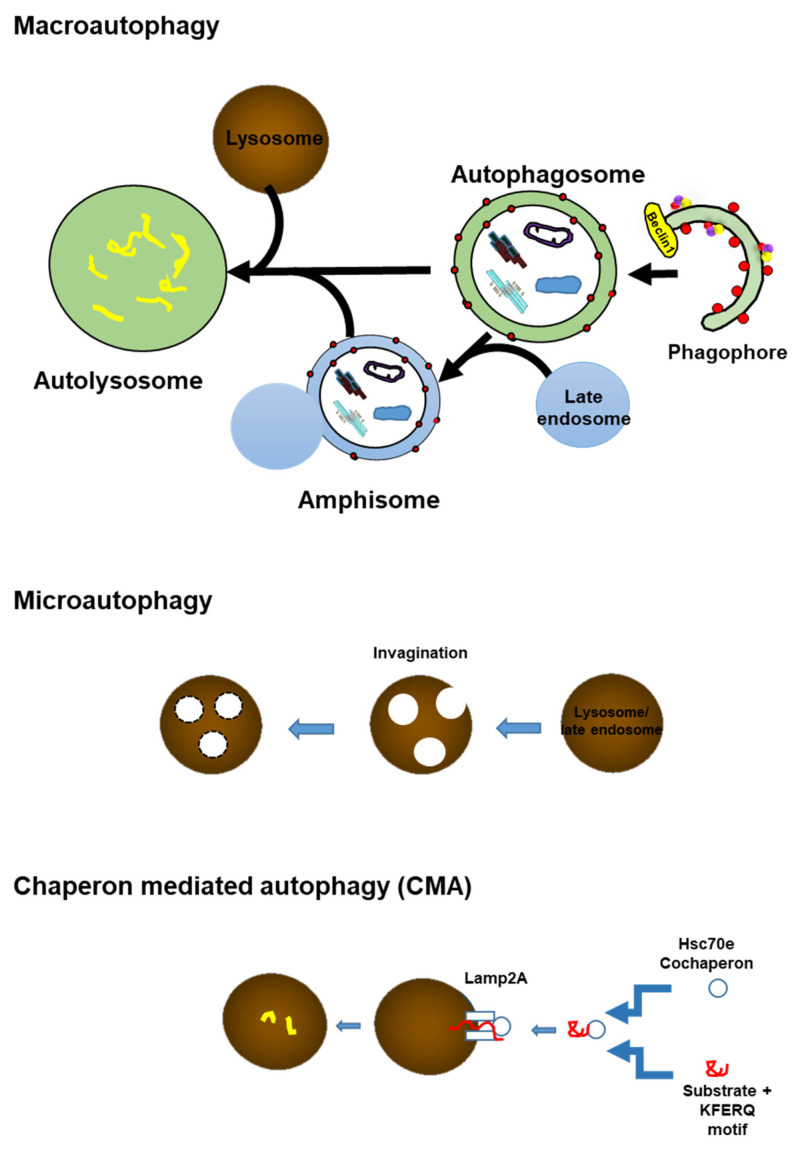
Scheme showing three types of autophagy; Macroautophagy, microautophagy, and the chaperon-mediated autophagy pathway. Macroautophagy: A part of the cytoplasm including the organelle is engulfed by an isolated membrane, the phagophore, which forms an autophagosome. By the fusion of the outer membrane with a lysosome, the internal materials are digested in the autolysosome. Microautophagy: A small amount of cytoplasmic material is trapped in the lysosome/vacuole through the random process of membrane erosion. Chaperon-mediated autophagy (CMA): Proteins with a KFERQ like motif are identified by cytosolic Hsc 70 and co-chaperons. Then, the substrate proteins are translocated to the lumen of the lysosomes after docking with the lysosome.

Autophagy (Greek for “self-eating”) is a highly conserved pathway and unique mechanism that degrades large organelles and proteins via lysosomes. Autophagy is divided into three types, macroautophagy, CMA, and microautophagy (Figure 3) [21]. The term autophagy usually means macroautophagy. Autophagy is primarily considered a pro-survival machinery. Under physiological conditions, cells perform low levels of autophagy to maintain homeostasis, termed basal autophagy. However, autophagy activity can be upregulated by variety of life threating events, such as hypoxia, nutrient depletion, exposure to reactive oxygen species (ROS), invasion of microorganisms, organelle damage, and excessive accumulation of aggregated proteins, including Aβ and tau.

**Figure 4:**
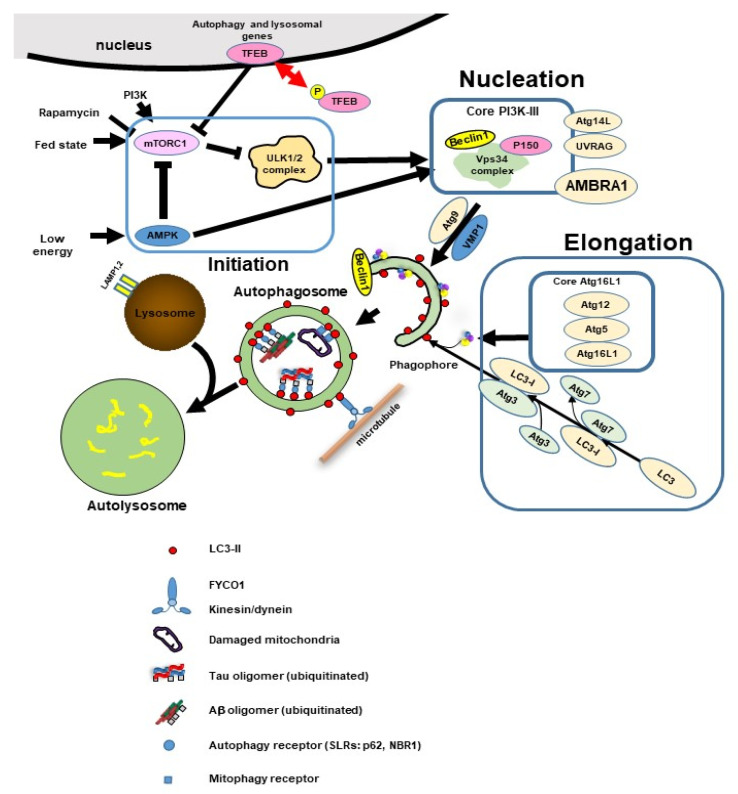
The sequence of autophagy in mammals. a. Initiation. In response to nutrient deprivation, AMPK phosphorylates mTORC1, releasing its inhibition on ULK, and activates the initiation complex. Downregulation of mTORC1 leads to the translocation of TFEB to the nucleus, where TFEB becomes upregulated, and various autophagy- and lysosome-related gene translation is done. ULK phosphorylates AMBRA-1, which promotes the interaction of Beclin-1 with p150 and PI3K-III (Vps34) in the PI3K-III complexes. UVRAG and Atg14L bind differently to form separate PI3K-III-complex populations in cells. AMBRA-1 also interacts with PI3K-III- complexes. B. Nucleation. ULK- and PI3K-III-complexes are translocated to early phagophore sites later with the help of Atg9 and VMP1, and local production of PI3P causes the recruitment of additional effectors and the first segregating membrane is formed. C. Elongation. Immediately after the activation of autophagy, Atg7 is upregulated, promoting Atg5-Atg12 dimer formation, which connects to Atg16L1 to form the Atg16L1 elongation complex. Atg7 also oversees the cleavage and lipidation of LC3 to LC3-I and then to LC3-II, which binds to the inside and outside of the formed phagosomal membrane. Mitochondria can be directly bound by mitochondrial receptors without poly-ubiquitination. In aggrephagy, poly-ubiquitinated protein aggregates (Aβ oligomer and tau oligomer) are engulfed by the phagophore via SLRs (sequestosome 1/p62-like receptors), which include P62 or NBR1. D. Maturation. Once the loaded phagophore is closed and the outer fraction of LC3-II is removed, the completed autophagosome fuses with the lysosome via a bonding process involving syntaxin members, and LAMP-1 and -2 creating an autolysosome. Autophagosomes are produced in the cytoplasm at random sites. The autophagosomes are delivered to lysosomes at the microtubule-organizing center. Transport of autophagosomes toward the plus end of microtubules involves the interaction of the LIR motif of FYCO1 with LC3-II on the outer membrane of the autophagosome. Abbreviations: PI3K-III: class III phosphatidylinositol 3-kinase; LC3-II: microtubule-associated protein light chain 3-II; LAMP-1, LAMP-2:lysosome-associated membrane protein 1, 2; SLRs: Sequestosome 1/p62-like receptors; FYCO1: FYVE and coiled-coil domain containing 1; p62: p62/sequestosome-1; NBR1: neighbor of BRCA1 gene 1; TFEB: transcription factor EB.

Many previous studies demonstrated that autophagy is highly selective [22]. Selective autophagy includes pexophagy (peroxisome), mitophagy (mitochondria), xenophagy (bacteria), and aggrephagy (misfolded proteins with aggregation) (Figure 4). The discovery of selective autophagy receptors in mammalian cells, along with studies on the cytoplasmic-to-vacuolar targeting pathway, helped to clarify some of the mechanisms of selective autophagy. Indeed, autophagy is highly dynamic, and its process is composed of initiation, nucleation, and elongation.

## 4. Overview of Autophagy

### 4.1. Autophagy Initiation

The ULK1(unc-51-like kinase 1) complex is considered to be an apical initiator of mammalian autophagy [23]. ULK1 and Atg (autophagy-related protein) 13 are core to the ULK1 complex and are further supported by Atg101 and FIP200 (focal adhesion kinase (FAK) family-interacting protein) scaffold protein. The ULK1 complex is disturbed by stress signaling pathways, including the mechanical target of rapamycin complex 1 (mTORC1) pathway (Figure 4). Under nutrient-rich conditions, mTORC1 binds to ULK1 and inhibits it. In contrast, intracellular amino acid depletion after starvation activates the energy level-dependent sensor AMPK (5’ adenosine monophosphate-activated protein kinase). AMPK phosphorylates mTORC1 and aids in the dissociation of mTORC1 from ULK. Downregulation of mTORC1 leads to the translocation of transcription factor EB (TFEB) to the nucleus, where TFEB becomes upregulated, and autophagy- and lysosome-related genes are translated. ULK1 forms complex with Atg13, FIP200, and Atg101 [23]. The fully bound ULK-1 complex, together with the class III phosphatidylinositol 3-kinase (PI3K-III) complex, translocates to the membrane compartment of the phagophore assembly (Figure 4) [24]. 

### 4.2. Phagophore Nucleation 

The lipid kinase PI3K-III (Vps34), its control subunit P150, and Beclin-1(Atg6) are the major constituents of the PI3K-III complex, which is essential for phagophore formation and maturation to autophagosome. Other important co-regulators of the PI3K-III complex are Atg14L, AMBRA-1 (autophagy and Beclin 1 regulator 1), and UVRAG (UV resistance-associated gene). Atg14L and UVRAG compete with Beclin-1 and constitute separate PI3K-III complexes. They are needed in autophagosome biosynthesis and maturation, and lysosomal fusion. At the nucleation site of the phagophore, the activated PI3K-III complex produces a local pool of phosphatidylinositol-3-phosphates (PI3Ps) (Figure 4).

### 4.3. Elongation and Closure 

The elongation of the new organelle relies on the constant uptake of membranous material into the expanding body. VMP1 (vacuole membrane protein 1)anchors PI3K-III complexes to the expanding surface of the phagophore by connecting to Beclin-1. Atg9 shuttles between intracellular membranous compartments (e.g., mitochondria, Golgi apparatus, or plasma membrane), transferring phospholipid fragments for incorporation into expanded structures. Ubiquitin-like proteins LC3 (microtubule-associated protein light chain 3) and the E3 ligase Atg12 are required for autophagosome expansion sites, together with associated ubiquitin-like binding systems. Atg7 upregulates Atg12 and Atg10 is responsible for binding Atg12 to Atg5. The Atg-12-Atg5 heterodimer then combines with Atg16L1 to form the core of the entire Atg16L1 complex. LC3-I is produced by cleavage of LC3 by cysteine protease Atg4B. LC3-I then migrates to the inner and outer membranes of the expanding phagophore by Atg7. LC3-I is passed to Atg3, which binds to phosphatidylethanolamine (PE) and becomes LC3-II through the local action of PI3K-III and the Atg16L1 complex, and is anchored to the membrane of autophagosomes (Figure 4). Transporter proteins move between membrane compartments in the cell (mitochondria, Golgi apparatus, plasma membrane, etc.) and transfer fragments of phospholipids for incorporation into expanded structures. Mitochondria can be captured by mitochondrial receptors directly without poly-ubiquitination. In aggrephagy, poly-ubiquitinated protein aggregates (Aβ and tau oligomers) are engulfed by the phagophore via SLRs (sequestosome 1/p62-like receptors), which include P62 (p62/sequestosome-1) or NBR1 (neighbor of BRCA1 gene 1).

Autophagosomes are formed at random sites in the cytoplasm. The autophagosomes are delivered to the microtubule-organizing center where lysosomes are enriched along microtubules [25]. Transport of autophagosomes toward the plus end of microtubules involves the interaction of the FYCO1 (FYVE and coiled-coil domain containing 1) with LC3-II on the outer membrane of autophagosomes [22]. 

### 4.4. Lysosomal Fusion

During the fusion of the fully developed autophagosome with a lysosome, the Atg16L1 complex dissociates from the vesicle, and cytosolic fractions of LC3-II are removed by Atg4B. Fusion of an autophagosome and lysosome requires the SNARE (soluble N-ethylmaleimide-sensitive factor activity part receptor) complex, involving several syntaxis and Atg14L1 [26]. This complex primes the autophagosome for the fusion step with the lysosome. Acidification and further maturation into matured autolysosomes take place with the help of Rab family, small GTPases and LAMP-1 and -2 (lysosome-associated membrane proteins 1 and 2). 

The inner membrane components of autolysosome are degraded by lysosomal hydrolases. A series of lysosomal proteases (cathepsins B, D, and L) are needed to degrade mammalian cells. When macromolecules are degraded in lysosomes, monomer molecules, including amino acids and lipids, are recycled into the cytoplasm for reuse. 

## 5. Disturbance of the Autophagy Lysosome System in AD and Related Disorders

### 5.1. Disturbance of the Autophagy Lysosome System in AD

In 1967, Suzuki and Terry discovered that in the AD brain, swollen neurites and dystrophic neurites surround the SPs [27]. In the dystrophic neurites, they noted many round or oval, laminar 0.5 μm diameter materials. However, at that time, their identity was not clear. In 2005, Nixon et al. revealed them to be autophagic vacuoles (Avs) [28]. Avs are composed of autophagosomes, amphisomes, and autolysosomes in the process of macroautophagy (Figure 3). Macroautophagy is constitutionally active and efficiently functions in young neuronal cells. However, in the AD brain, the function of macroautophagy is disturbed. 

Autophagy induction was observed under conditions of cell stress [29] and neurodegeneration, including AD [30]. Macroautophagy induction and disturbance are observed in the AD brain, and in mice overexpressing mutant human presenilin 1 (PS1) and amyloid precursor protein (APP), which causes Aβ-containing Av accumulation in affected neurons [28]. In PS1-APP mice, autophagosomes proliferate in dendrites at a young age before Aβ is deposited. This suggests that macroautophagy induction is an early event in AD, and not a consequence of Aβ deposition [31]. Boland et al. reported that when mTOR kinase activity was suppressed by rapamycin or nutrition was withdrawn, cathepsin D-positive autophagosomes, but not LC3-II-positive autophagosomes, predominated in primary cortical neurons. Thus, autophagosome clearance is efficient in neurons. On the contrary, late step impairment of macroautophagy by lysosomal protease inhibition or vinblastine treatment causing microtubule impairment leads to Av accumulation in primary cortical neurons, which resembled Avs observed in dystrophic neurites in the AD brain and AD mouse model. Thus, the authors stated that the autophagic pathology noted in AD brains is likely due to disturbed clearance of Avs rather than the induction of autophagy [32]. 

PS1 is required for the turnover of protein substrates in lysosomes for autophagy and endocytosis; loss of PS1 leads to impaired acidification of autolysosomes and inhibition of cathepsin (cysteine cathepsins are proteases responsible for proteolysis in lysosomes) activation, resulting in autophagy dysfunction [31]. Therefore, mutations in PS1, which are the major cause of FAD, are directly related to the mechanisms that promote the pathogenesis of AD. Deletion of PS1 inhibits protein degradation by macroautophagy [33].

### 5.2. Sporadic Inclusion Body Myositis and Tau and Aβ 

Sporadic inclusion body myositis (s-IBM) is the most common progressive muscular disorder in the elderly in Western countries. S-IBM patients exhibit severe disability and no effective treatment has been developed. The pathological hallmarks of s-IBM are Avs and accumulated ubiquitin-positive multiportion aggregates that include Aβ or phospho-tau with a β-sheet structure. Endoplasmic reticulum stress (ERS) and inhibition of 26S proteasomes are also associated with s-IBM, and are thought to exacerbate the deposition of misfolded proteins. Nogalska et al. reported that in s-IBM, cathepsin D and B, lysosomal enzymes, are inactivated. The authors also detected the presence of LC3-II and reduced mTORC1-mediated phosphorylation of p70S6 kinase, which suggests increased autophagosome maturation. It was concluded that in s-IBM, lysosomal proteolysis was inactivated by muscle, which may exacerbate misfolded protein deposition regardless of increased autophagosome maturation, and that ERS induces s-IBM-defective lysosomal function [34]. Disturbances of mitophagy were also reported in s-IBM pathology, suggesting the pathogenic importance of abnormal mitophagy in s-IBM [35]. 

### 5.3. Tau and Aβ Can Induce Autophagy Dysfunction

Previous studies revealed that phosphorylated tau protein and Aβ disturb autophagy and mitophagy, which are the major events in AD pathogenesis [36,37]. Age-dependent increases in phosphorylated tau and Aβ reduce autophagy and mitophagy proteins. Aberrant interactions between phosphorylated tau and Drp-1 and Aβ and PINK1 (PTEN-induced kinase 1)/parkin induce the inability to remove defective mitochondria and other debris from neuronal cells [36]. Feng et al. also found that tau accumulation inhibits autophagosome–lysosome fusion by inhibiting the expression of IST1 (IST1 factor associated with ESCRT-III), which in turn inhibits ESCRT-III (endosomal sorting complex required for transport-III) formation, revealing a vicious circle of tau deposition and autophagy impairment in the chronic progression of AD [38].

FKB52 is a member of the immunophilin protein family, which functions in immunomodulation and basic cellular processes, including protein folding and transport. The encoded protein is a cis-trans prolyl isomerase that connects to the immunosuppressants FK506 and rapamycin. The immunophilin FKBP4/FKBP52 was previously reported to localize to the lysosomal system of healthy human neurons, suggesting that it is involved in lysosomal function [39]. Truncation of tau at the C terminus is essential for tau aggregation [18,19,20] (Figure 1 and Figure 2). This caspase3 cleaved tau (D421) is colocalized with the FKB52 in the autophagy endolysosome system in AD neurons. The amount of FKBP52 is markedly low in the AD brain, and the reduction of FKBP52 correlates with the abundance of NFT. Autophagy inhibition in Tau-inducible neuroblastoma resulted in the release of FKBP52 into the extracellular environment. In the AD brain, FKBP52 is aberrantly released from NFT-negative neurons, suggesting that it is associated with the extent of early Tau-D421 accumulation in neurons [40]. This implies that caspase cleavage of tau can be the trigger of autophagy inhibition via FKBP52 release, or vice versa.

## 6. Tau Degradation Pathway

In the AD brain, 20S proteasomal (core particle) activity, especially trypsin-like activity, is disturbed [41]. If tau is ubiquitinated by the CHIP, it will be degraded by proteasomes [42]. Proteasomes can degrade endogenous tau regardless of whether it is full-length or a truncated species. However, PHF-tau cannot be degraded by proteasomes and PHF tau disturbs the proteasomes. 

**Figure 5:**
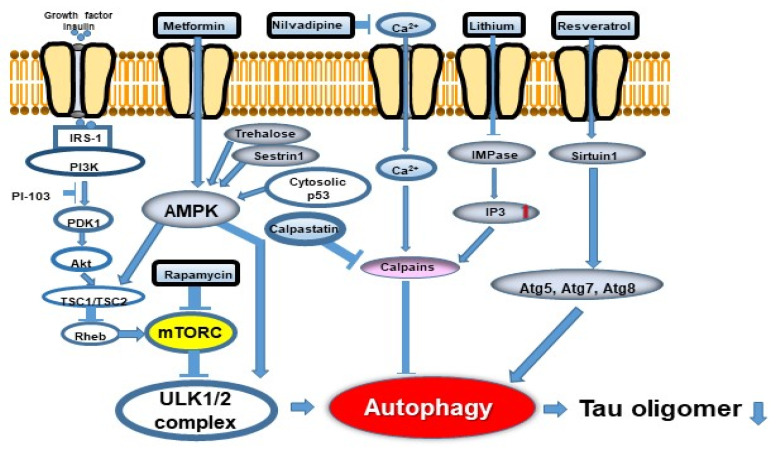
Autophagy flux and potentially effective drugs. Two signaling pathways, mTORC1-dependent and -independent, are involved in autophagy regulation. Abbreviations: mTORC1: mechanistic target of rapamycin 1; IRS-1: insulin receptor substrate-1; PI3K: phosphoinositide 3-kinase; SIRT1: sirtuin 1 (revised from Curr. Alzheimer Res. 2018, 15, 1283-1296 [25].

The relationship between tau degradation and the autophagy lysosome system was unclear for a long time. Chloroquine, a drug for malaria, impairs autophagy by disturbing autophagosome fusion with lysosomes rather than by perturbing the acidity and/or digestive function of lysosomes [43]. In a study on chloroquine myopathy, it was discovered that tau accumulated in rat muscle [44]. This implies that tau can be degraded by a lysosome autophagy system. We investigated this using a neuronal cellular model of tauopathy, M1C cells that harbor wild-type tau (4R0N) by tetracycline-off induction [4,5,20,45,46,47,48]. M1C cells were treated with the lyososomotrophic agent NH4Cl and autophagy inhibitors chloroquine and 3 methyladenine (3MA). All chloroquine NH4Cl and 3MA treatment increased tau accumulation in the cells. Chloroquine treatment increased the accumulation of AVs observed by electron microscopy [46]. The following year, Wang et al. demonstrated that tau degradation is through macroautophagy [49]. However, they also found that N terminus truncated species are preferentially trapped by CMA, and C terminal truncation occurred. The C terminal-truncated tau promoted tau aggregation [6]. Later, rapamycin, which induces autophagy (Figure 4 and Figure 5, Table 1) was reported to reduce aggregated tau [50]. Therefore, the autophagy lysosome system degrades not only soluble tau but also insoluble, aggregated tau. C terminal-truncated tau is preferentially digested though autophagy. However, full-length tau is preferentially digested through proteasomes [51]. Recently, it was proposed that a large fraction of tau is degraded by CMA, and upon acetylation, tau is preferentially degraded by macroautophagy and endocytic microautophagy [52].

As mentioned above, caspase-3 activation increases tau truncation at the C terminus [6,17,18,19,20] and tau aggregation, leading to autophagy dysfunction [40]. Although further studies are needed to provide direct evidence, caspase-3 activation may be the cause of neurodegeneration, including AD.

## 7. Mitophagy and Tau

Defective mitochondria accumulation is a hallmark of normal aging and age-related neurodegenerative diseases, including AD. Mitophagy is impaired in iPS cell-derived human AD neuronal cells and in AD animal models. In Caenorhabditis elegans (C-elegans) models, stimulation of mitophagy restores cognitive disturbances via PINK-1, PDR-1 (Parkinson’s disease-related-1; parkin)-, or DCT-1 (DAF-16/FOXO-controlled germline-tumor affecting-1)-dependent pathways. Mitophagy reduces aggregated Aβ42 and Aβ40, and inhibits cognitive disturbances in APP/PS1 mice through microglial phagocytosis of Aβ in SPs and suppressing inflammation of the neuron. Mitophagy activation also inhibits hyperphosphorylation of tau in human neurons and restores cognitive disturbances in transgenic C. elegans and mice models [53]. Cummins et al. also reported that both human wild-type tau (hTau) and mutant tau (hP301L) observed in FTDP-17 disturb mitophagy in a neuroblastoma cell line by decreasing the mitochondrial translocation of Parkin. H-tau expression in the nervous system itself impaired mitophagy, and the expression of hP301L resulted in complete mitophagy suppression in C. elegans [54].

## 8. Diabetes and Tau and Autophagy 

Epidemiological studies have revealed that the prevalence of AD is two-fold in people with diabetes [55]. Insulin signaling impairments are associated with enhanced NFT and SP deposition in the AD brain [56]. Diabetic model animals also exhibit memory impairment and pathological changes of AD, including phosphorylated tau [57]. Streptozotocin-induced diabetic rats demonstrated impaired performance in several cognition tests, including the Morris water maze compared with control animals. Furthermore, diabetes-induced AD-like behavioral and pathological findings, including Aβ and phosphorylated tau deposition, were associated with decreased autophagy in neurons. Disturbed cell autophagy was also reproduced in neuronal cultures in high glucose conditions [58]. Chen et al. reported that metformin ameliorated cognitive decline in a rodent diabetes model, reduced hyperphosphorylated tau, and restored the disturbed autophagy in diabetic animals, all of which were reversed by inhibition of autophagy. In cultured HT22 cells in high glucose conditions, metformin promoted autophagy in a dose-dependent manner via AMPK (Figure 5) [59].

## 9. Tau Propagation Due to Autophagy Impairment 

### 9.1. The Secretion Pathways of Pathological Tau Protein

**Figure 6:**
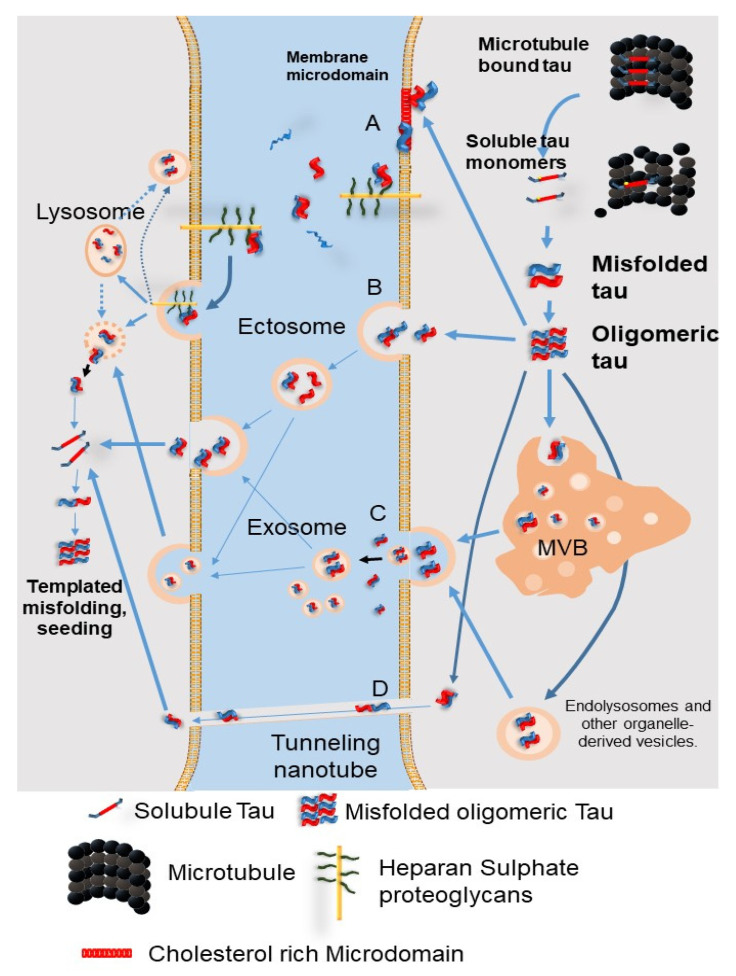
The secretion pathways of pathological tau protein. (**A**) Direct secretion of tau through the plasma membrane involves clustering of tau at the plasma membrane, interaction of cholesterol/sphingomyelin-rich membrane microdomains with specific lipids, permeation into the plasma membrane, and release from the plasma membrane by cell surface heparin sulfate proteoglycans. (**B**) Tau is secreted into ectosomes, which are larger and have a different composition than the exosomes released from the plasma membrane. Both ectosomes and exosomes function in the same manner after they are released from the cell, fusing to the target cell or being endocytosed. (**C**) Secretion of tau by exosomes and organelle hitchhiking. Tau may be secreted when the membrane of late endosomes exits inward to form luminal vesicles of the multivesicular body (MVB), which are packed into exosomes, and when the MVB membrane fuses with the plasma membrane. Organelle hitchhiking pathways that may be involved in the secretion of tau and other misfolded cytoplasmic proteins include secretory endolysosomes associated with the autophagy–lysosome pathway. The misfolding-associated protein secretion (MAPS) pathway involves chaperone-mediated uptake of misfolded cytoplasmic proteins into the endoplasmic reticulum, followed by fusion of endolysosomal vesicles with the plasma membrane and secretion, thereby releasing vesicle-free tau into the extracellular space. (**D**) Tau seeds are transferred between cells via tunnel-like nanotubes that directly connect the cytoplasm of two adjacent cells. MVB: multivesicular bodies. (Revised from Brunello, C.A. et al., *Cell Mol. Life Sci.* **2020**, *77*, 1721-1744 [60]).

Although tau is a microtubule-associated protein observed in the cytosol, it is also found outside of the cells physiologically. Misfolded tau can be transferred between cells through four non-exclusive mechanisms (Figure 6): (A) Direct secretion of tau through the plasma membrane involves clustering of tau at the plasma membrane, interaction of cholesterol/sphingomyelin-rich membrane microdomains with specific lipids, permeation into the plasma membrane, and release from the plasma membrane by cell surface heparin sulfate proteoglycans. (B) Tau is secreted into ectosomes, which are larger and have a different composition than the exosomes released from the plasma membrane. Both ectosomes and exosomes function in the same manner after they are released from the cell, fusing to the target cell or being endocytosed. (C) Secretion of tau by exosomes and organelle hitchhiking. Tau may be secreted when the membrane of late endosomes exits inward to form luminal vesicles of the multivesicular body (MVB), which are packed into exosomes, and when the MVB membrane fuses with the plasma membrane. Organelle hitchhiking pathways that may be involved in the secretion of tau and other misfolded cytoplasmic proteins include secretory endolysosomes associated with the autophagy–lysosome pathway. The misfolding-associated protein secretion (MAPS) pathway involves chaperone-mediated uptake of misfolded cytoplasmic proteins into the endoplasmic reticulum, followed by fusion of endolysosomal vesicles with the plasma membrane and secretion, thereby releasing vesicle-free tau into the extracellular space. (D) Tau seeds are transferred between cells via tunnel-like nanotubes that directly connect the cytoplasm of two adjacent cells. Regardless of the secretory pathway, aggregated tau will eventually reach the cytoplasm of receptor cells and cause templated misfolding. The misfolded protein acts as a conformational template, turning a normal protein into a pathogen. At present, it is unclear which of the above mechanisms are involved in tau synaptic release, and whether the same mechanism is responsible for physiological tau synaptic release and pathological tau synaptic release [60].

### 9.2. Tau Propagation Due to Autophagy Impairment

Tau propagation through neurons is considered important for the progress of tau-mediated neurodegeneration. It was previously reported that tau is acetylated by the lysine acetyltransferase p300/ CBP, and the degradation and toxicity of tau are also controlled by p300/CBP [61]. P300/CBP, which is abnormally upregulated in tauopathies, disturbs the autophagy lysosome pathway, which leads to the excessive secretion of tau [62]. A new p300 inhibitor, SMDC37892, increased autophagic flux and reduced tau secretion in a mouse model of tauopathy and in human and rodent neocortex culture. In contrast, highly activated p300/CBP blocked the autophagic flux and enhanced tau secretion in neurons [62]. Tang et al. also reported that activated mTORC, which leads to autophagy inactivation, promotes tau secretion into the extracellular space in an exosome-independent manner in neuroblastoma cells, suggesting that tau release is mediated by mTORC via an exosome-free route (Figure 6) [63]. Recently, it was also reported that CMA blockade upregulated cell to cell propagation in a rodent model of tauopathy [52].

To the contrary, autophagy inducers increased both total and phosphorylated tau secretion, which was reduced by Beclin1 knockdown or autophagy inhibitors in a hTau-overexpressing human neuroblastoma cell line [64]. In addition, six isoforms of tau are secreted in an autophagy-dependent manner (Figure 6) [64]. Thus, this issue remains controversial. 

## 10. Possibilities of Autophagy Modulators as a Therapeutic Approach to AD

Autophagy regulation may be a therapeutic strategy for AD or a method to delay its progression. Autophagic pathways and autophagy-modulating drugs are described in Figure 5. Autophagy inducers are usually divided into two major groups, mTORC-dependent and mTORC-independent. The mTORC-dependent autophagy inducer rapamycin reduced NFT in P301S transgenic mice [50] and phosphorylated tau in zebrafish with A152T tau [65]. The rapamycin analogue temsirolimus reduced the amount of tau [66]. Congdon et al. reported that methylene blue induced autophagy and attenuated the sarkosyl insoluble tau accumulation in vitro and in vivo via mTORC1 suppression [67]. ATP-competitive mTORC inhibitors OSI-027 and AZD2014 reduced highly-phosphorylated and aggregated tau significantly more than rapamycin in patient iPSC-derived neuron models [68]. As a result, the tau-mediated neuronal stress vulnerability was reduced. The authors found that mTORC inhibition and autophagy upregulation directly lead to tau clearance [68]. The ATP-competitive mTORC1/2 blocker PP242 also reduced the amount of tau.

In addition to mTORC-dependent autophagy inducers, the AMPK activator metformin reduced tau accumulation [59]. Another AMPK activator trehalose also reduced endogenous tau in primary neurons via autophagy activation in an mTORC-independent manner [69]. Of note, phosphorylation of tau at several sites in the AD brain has little influence on its degradation by autophagy. Moreover, autophagy upregulation suppresses tau aggregation and reduced its cytotoxicity [69]. Trehalose also reduced the amount of insoluble tau in P301S mice [70]. The IMPase inhibitor lithium [71] was reported to activate autophagy and reduce tau accumulation in an mTORC-independent manner. Autophagy inducers, the imidazoline receptor agonists clonidine and rilmenidine, were effective in a zebrafish model harboring A152T tau [65]. Curcumin was reported to reduce the amount of dimeric tau in the soluble fraction in hTau mice via autophagy activation [72]. The authors hypothesized that this was due to increased membrane-bound Hsc70 by curcumin, which is required for tau degradation in CMA (Figure 3) [72]. Wang et al. reported that curcumin downregulated the PI3K/Akt/mTORC pathway [73]. The small molecule TFEB activator, curcumin analogue C1, reduced tau and Aβ pathology via autophagy activation [74]. Binder et al. reported that optical induction of TFEB under blue light attenuated AD pathology, including phosphorylated tau in neuronal cells and AD patient-derived iPSC cells [75]. The HSPA/HSP70 co-chaperone BCL2-associated athanogene 3 (BAG3) promotes endogenous tau removal via autophagy. BAG3 is important in autophagic flux in the axons of matured neuronal cells by interacting with the post-synaptic cytoskeleton protein synaptopodin (SYNPO). Deletion of BAG3 or SYNPO inhibits the fusion of autophagosomes and lysosomes mainly in the post-synaptic section. Blocking autophagy induces deposition of p62 and phosphorylated tau at Ser262 (p-Ser262). Moreover, phosphorylated tau is deposited in autophagosomes at post-synaptic sections [9]. Rifampicin-treated tau mice had less oligomeric tau deposition, hyperphosphorylated tau, synaptic loss, and activated microglia in a dose-dependent manner, and their cognition almost completely recovered. Furthermore, rifampicin reduced p62 in the brain, not influencing LC3 conversion, suggesting the recovery of lysosomal function [76]. Rifampicin was also reported to increase ATP6V0A1, which promotes lysosomal function [77].

Nicotinamide treatment improved mental decline and Aβ and hyperphosphorylated tau deposition in 3xTgAD mice. Nicotinamide increased NAD+, increased the resistance of mitochondria against oxidative stress, and promoted the autophagy–lysosome process [78]. Huuskonen et al. revealed that the FDA-approved drug for skin lymphoma bexarotene alters autophagy markers and reduces autophagic flux in neurons harboring P301L-Tau. Bexarotene also improved mitochondrial disturbances in P301L-Tau neuronal cells [79]. Lonafarnib, a farnesyl transferase inhibitor, reduced tau inclusions, behavioral abnormality, brain atrophy, and microgliosis in rTg4510 tauopathy mice [80]. The tyrosine kinase inhibitors nilotinib and bosutinib caused phosphorylated tau degradation through autophagy and restored the neurotransmitter imbalance in P301L mice [81]. 

Menzies et al. reported that calpain inhibition via siRNA-activated autophagy and ameliorated tau induced toxicity in a Drosophila model [82]. Smith et al. reported that the micro RNA, miR-132/212 reduces tau in mice [83]. Conversely, miRNA-132/212 depletion increased aggregated tau by suppressing autophagy [84]. 

Our study suggested that Rho-ROCK inhibitors markedly reduce oligomeric tau and activate autophagy based on a cell culture model of tauopathy (M1C cells) and mouse model of tauopathy (rTg4510), although the autophagy-activating pathway remains unclear [48] (Table 1).

**Table 1:** Autophagy inducers that may be effective against Alzheimer’s disease. Abbreviations: mTORC: mechanistic target of rapamycin complex; AMPK: adenosine monophosphate-activated protein kinase; cAMP: cyclic adenosine monophosphate; IMPase: inositol monophosphatase; TFEB: transcription factor EB.

Autophagy Target Drugs	Action	References
mTORC dependent autophagy inducer		
Rapamycin	mTORC1 inhibitor	[50,65]
Rapamycin analogues	mTORC1/2 inhibitor	[66,68]
CCI-779, RAD001 (everolimus), AP23573		
Selective ATP-competitive small molecule		
PP242, OSI-027, AZD2014 and AZD8055		
Methylene blue	mTORC inhibitor	[67]
Curcumin	mTORC inhibitor	[73]
**Other autophagy modulating drugs**		
**mTORC independent autophagy inducer**		
Metformin	AMPK activator	[59]
Trehalose	AMPK activator	[69]
Lithium	IMPase inhibitor (Reduce inositol and IP3)	[71]
Clonidine, rilmenidine	Imidazoline receptor agonists (Reduce the cAMP)	[65]
Curcumin	Induce Hsp70 involved in CMA	[72]
Curcumin analogue (C1)	TFEB activation	[74]
BAG3	Promotion of glutamine consumption and glutaminolysis	[9]
Nicotinamide	Enhancing lysosome/autolysosome acidification	[78]
	Reduce autophagosome accumulation	
Rifampici	Elevate ATP60V01	[76,77]
P300/CBPinhibitor (SMDC37892)	Atg5,7,8,12 stabilization	[62]
Lonafenib	Falnesylation inhibitor	[80]
**Unknown**		
Bexarotene	Restores defective mitochondrial respiration in P301L-tau mutation	[79]
Nilotinib, bosutinib	Tyrosine kinase inhibitor	[81]
Rho-ROCK inhibitor	Rho-ROCK inhibition	[48]
**Gene therapy**		
MicroRNA	Increased clearance of tau in AD mice	[83,84]
siRNA(calpain knockdown)	Inhibit calpain activation	[82]
Optogenetic TFEB inducer	TFEB induction	[75]

Although activation of autophagy is a promising therapeutic approach, over-activation of autophagy in neurodegenerative diseases where lysosomal clearance is impaired may promote pathogenesis. In other words, the success of autophagy-based therapy depends on lysosome clearance [85]. Furthermore, the effects of autophagy activation can vary greatly depending on the physiological status of the cell, especially in response to proteotoxic stress and aging. In order to increase the effectiveness of autophagy, strategies to prevent or reverse the disruption of specific processes that have been disrupted are now considered to be important [85].

## 11. Caspase Activation and Autophagy

Autophagy is constantly active in the central nervous system [32] and helps to maintain homeostasis by removing abnormal proteins and organelles, preventing the accumulation of aggregated proteins, thereby supporting energy demand and maintaining neuronal plasticity. There is evidence that autophagy has neuroprotective effects and is especially important in neurons, which are post-mitotic cells.

Autophagy has a strong relationship with programmed cell death (apoptosis), which is initiated primarily by mitochondrial membrane permeabilization (MMP). When only a few mitochondria are MMP-initiated, depolarized mitochondria are selectively removed by autophagy, i.e., mitophagy occurs. However, when the threshold for mitophagy is exceeded, MMP becomes a fatal event. When the apoptosis regulatory protein Bcl-2 is released from the activated autophagy protein complex, these molecules are thought to block the apoptotic pathway. Conversely, induction of apoptosis is considered to be associated with the inactivation of autophagy. For example, caspase-3 inhibits the activity of autophagy by cleaving Beclin-1, and the C-terminal fragment of Beclin-1 produced by this cleavage leads to the amplification of mitochondria-mediated apoptosis.

Although autophagy is generally thought to be a survival-promoting mechanism, autophagy and apoptosis are interdependent. It is noteworthy that apoptosis and autophagy share common regulators, such as Beclin-1, Bcl-2, p53, and Atg5, which may interact to promote neuronal cell death. When autophagy is inhibited, neuronal apoptosis is promoted [86]. Autophagy also promotes cell death during cell elimination and neuronal excitotoxicity as a result of overactivation. Thus, several studies demonstrate that autophagy inhibition leads to increased neuronal survival in the case of hypoxic/ischemic brain injury in mice and necrotic cell death in the case of C. elegans.

The function of autophagy in cell death and its detailed mechanisms remain unknown, and it is not clear whether autophagy-induced cell death is associated with apoptosis or whether it is a separate process [85].

## 12. Conclusions

As discussed above, dysfunction of autophagy, which disrupts the effective clearance of misfolded proteins and cytoplasmic oligomers, has been observed in many neurodegenerative disorders, including AD, Parkinson’s disease, amyotrophic lateral sclerosis, and Huntington’s disease. Therefore, regulation of autophagy may be favorable in the prevention and therapeutics of AD by preventing tau aggregation. However, as mentioned in the previous section, crosstalk between autophagy and caspases has been reported [86]. Caspases can increase autophagy under certain conditions [87]. Autophagy has been reported to be a “double-edged sword”. While autophagy protects cells from apoptosis and necrosis by degrading toxic substances, abnormal activation of autophagy can lead to autophagic stress, a relatively persistent state of imbalance in which the rate of autophagosome vacuole formation exceeds the rate of autophagosome vacuole degradation [88]. Previous studies have proven that autophagy plays a dual role in neurodegenerative diseases: in the early stages of AD, autophagy is enhanced, facilitating the clearance of abnormally folded proteins and preventing further progression of AD [88]. On the other hand, in the advanced stage of the disease, autophagy is in a state of autophagic stress, where the clearance of autophagosomes cannot keep up with the formation of autophagosomes. Therefore, proper activation of autophagy is essential for the therapeutics of tauopathies, including AD [85]. 

## Abbreviations

NFT	Neurofibrillary tangle
SPs	senile plaques
AD	Alzheimer’s disease
GSK3β	glycogen synthase kinase 3β
cdk5	cyclin dependent kinase5
PP2A	protein phosphatase 2A
mTORC	mechanistic target of rapamycin complex
Atg	autophagy related protein
ULK-1	Uncoordinated 51-like autophagy activating kinase 1
PI3K-III	class III phosphatidylinositol 3-kinase
Avs	autophagic vacuoles
LC3	Microtubule-associated protein light chain 3
AMPK	adenosine monophosphate-activated protein kinase
P62	p62/sequestosome-1
FYCO1	FYVE and coiled-coil domain containing 1
TFEB	transcription factor EB
